# Effect of Liquid Nitrogen Freezing Temperature on the Muscle Quality of *Litopenaeus vannamei*

**DOI:** 10.3390/foods12244459

**Published:** 2023-12-13

**Authors:** Wenda Yan, Qinxiu Sun, Ouyang Zheng, Zongyuan Han, Zefu Wang, Shuai Wei, Hongwu Ji, Shucheng Liu

**Affiliations:** 1 College of Food Science and Technology, Guangdong Ocean University, Guangdong Provincial Key Laboratory of Aquatic Product Processing and Safety, Guangdong Province Engineering Laboratory for Marine Biological Products, Guangdong Provincial Engineering Technology Research Center of Seafood, Key Laboratory of Advanced Processing of Aquatic Product of Guangdong Higher Education Institution, Zhanjiang 524088, China; ywd7522@163.com (W.Y.); zhengouyang07@163.com (O.Z.); longnv0206@163.com (Z.H.); wangzefugdou@163.com (Z.W.); weishuaiws@126.com (S.W.); jihw62318@163.com (H.J.); 2 Collaborative Innovation Center of Seafood Deep Processing, Dalian Polytechnic University, Dalian 116034, China

**Keywords:** *Litopenaeus vannamei*, liquid nitrogen freezing, temperature, ice crystal, muscle quality

## Abstract

The implications of different liquid nitrogen freezing (LNF) temperatures (−35 °C, −65 °C, −95 °C, and −125 °C) on the ice crystal and muscle quality of white shrimp (*Litopenaeus vannamei*) were investigated in this essay. The results showed that better muscle quality was maintained after LNF treatment compared to that after air blast freezing (AF) treatment. As the freezing temperature of liquid nitrogen decrease, the freezing speed accelerated, with the freezing speed of LNF at −125 °C being the fastest. However, an excessively fast freezing speed was not conducive to maintaining the quality of shrimp. Among all the freezing treatments, LNF at −95 °C led to the lowest thawing losses and cooking losses, and the highest L* values, indicating that LNF at −95 °C could keep the water holding capacity of frozen shrimp better than that with other freezing methods. At the same time, LNF at −95 °C resulted in higher water holding capacity, and hardness values for shrimps than those with other frozen treatments (*p* < 0.05). In addition, the results of the water distribution of shrimps showed that treatment with a −95 °C LNF reduced the migration rate of bound and free water. Meanwhile, the microstructural pores of shrimps in the −95 °C LNF group were smaller, indicating that the ice crystals generated during −95 °C LNF were relatively smaller than those generated via other frozen treatments. In conclusion, an appropriate LNF temperature (−95 °C) was beneficial for improving the quality of frozen shrimp, and avoiding freezing breakage.

## 1. Introduction

*Litopenaeus vannamei* is also known as the South American white shrimp because of its fast growth, adaptability, tender meat, nutritiousness, and popularity. Its importance in agricultural production, as one of the aquatic resources with economic representation is emphasized [1]. Due to its high protein and water content, as well as the large number of endogenous enzymes and microorganisms in the shrimp, shrimp is prone to spoilage and a short shelf life [2]. Freezing technology is commonly used in the food industry, and can inhibit the spoilage caused by endogenous enzymes and microorganisms, allowing for the maximum retention of nutrients and the flavor of frozen food. In the course of the freezing procedure, the freezing temperature and speed affect the size, shape, and formation of ice crystallines formed in food. The size and distribution of ice crystals formed during freezing determine the quality of the frozen food [3]. Generally, slow freezing results in larger ice crystals and greater damage to the cell structure, leading to much juice loss, and a significant loss of quality after thawing.

Liquid nitrogen freezing (LNF) is a freezing method that utilizes a strong heat exchange between liquid nitrogen vaporization and food, which makes the temperature drop rapidly, and realizes quick freezing. The ice crystals formed via the rapid freezing of liquid nitrogen are small and evenly distributed, causing low damage to the cellular structure, and maintaining better quality after thawing. At the same time, due to the colorless and transparent nature of liquid nitrogen itself, the chemical properties are relatively stable, safe, and non-polluting. In recent years, a variety of liquid nitrogen refrigeration devices in the food industry have been vigorously promoted for use [4]. It has now been found that proper LNF can effectively improve the quality of frozen blueberries, hairtail, catfish, and other foods. Ketata et al. [5] reported that blueberries pretreated with LNF could maintain better quality. Liquid nitrogen technology has also been used more frequently in aquatic products. Hu et al. [6] found that mechanical damage in LNF hairtail (*Trichiurus haumela*) was much less than that in cold air-frozen hairtail. It has also been found that channel catfish fillets frozen via liquid nitrogen immersion showed less of a change in quality, a more compact fiber arrangement, less thawing loss, and a better retention of taste active compounds [7].

Although LNF technology has some of the above-mentioned advantages, it also has some shortcomings. When the temperature of liquid nitrogen is too low, it can cause the low-temperature fracture of the food, resulting in a loss of quality. For example, Lv et al. [8] found that below −100 °C, LNF led to cracks and fractures in cuttlefish, which affected its sensory evaluation. Teng et al. [9] found that oysters improved muscle quality, and maintained their flavor characteristics after being frozen at a −80 °C liquid nitrogen temperature. Yang et al. [10] found that golden pompano could maintain better muscle quality after being frozen at a −95 °C liquid nitrogen temperature. Therefore, the study of the optimal LNF temperature is crucial for food products, and the optimal temperature varies from one food product to another due to differences in moisture content, nutrient mechanisms, and tissue structures.

At present, the optimum LNF temperature for shrimp is not yet known. Therefore, the effect of different liquid nitrogen temperatures (−35 °C, −65 °C, −95 °C, and −125 °C) on the muscle quality of vannamei shrimp was investigated; meanwhile, ice crystal size and distribution during different LNF processes were also evaluated to search for the right LNF temperature for shrimp.

## 2. Materials and Methods

### 2.1. Chemicals

Methane (purity ≥ 36.0%), glacial acetic acid (purity ≥ 99.5%), ethanol (purity ≥ 99.7%), xylene (purity ≥ 99.0%), chloroform (purity ≥ 99.0%), neutral gum, and hematoxylin-eosin solution were used. All chemicals and reagents were of analytical grade, and were purchased from Solarbio Company (Beijing, China).

### 2.2. Preparation of Shrimps

Live non-pregnant *Vannamei Litopenaeus* of a uniform size (approximately 40 per kg) were bought from Dongfeng Fishery Market (Zhanjiang, China), and carried to the lab in oxygenated water. The shrimps were killed via anesthesia with crushed ice. The surface of the samples was blotted dry with filter paper and sealed in separate polypropylene sealing bags. All samples were randomly divided into 6 aliquots, and transferred to a 4 °C refrigerator for 12 h. Untreated fresh shrimps were used as a negative control group, while the other five groups were frozen at different liquid nitrogen temperatures (−35 °C, −65 °C, −95 °C, and −125 °C), and via air blast freezing (AF). For LNF, shrimp samples were placed on trays in the cavity of the liquid nitrogen quick-freezer (DJL-QF60, Shenzhen, China) for freezing, when the core temperature of the samples reached −18 °C, the samples were transferred to the −18 °C freezer cabinet to await measurement. For the AF group (the positive control), shrimps were placed in a freezer (BD/BC-768DKEMA, Hefei, China) at −35 °C until the central temperature of the samples reached −18 °C, and then the samples were transferred to a −18 °C freezer to await measurement.

The shrimp samples that had been subjected to both LNF and AF were stored in a refrigerator at −18 °C for one day prior to testing. The whole shrimp samples were used to measure the freezing curve and thawing loss, and for magnetic resonance imaging, and low-field nuclear magnetic resonance imaging; decapitated shrimp samples were used to measure cooking loss, and the second abdominal segments of the shrimp samples were used to test water holding capacity, color, hardness, and microstructure.

### 2.3. Freezing Curve

The freezing curve of the LNF and AF groups was determined via the use of a multiplex temperature tester (AT4500, Changzhou, China). This was performed as follows: the shrimp were transferred from the 4 °C refrigerator to the appropriate freezing environment, and the temperature probe of the multiplex temperature tester was inserted into the center of the second meat of the shrimp. After starting freezing, the change in temperature was recorded every second until the core temperature of the shrimp reached −18 °C (when freezing was completed). From the data recorded using the instrument, the freezing curve was plotted using Origin 2023 software (OriginLab, Northampton, MA, USA).

### 2.4. Thawing Loss

The method for measuring thawing loss was based on that of Atani et al. [11] with slight modifications. The mass of the sample weighed before thawing was represented by *M*_0_. Shrimp was placed in a 4 °C refrigerator to thaw until the central temperature of the shrimp body increased to 4 °C. The shrimps were then cleaned with surface water using dehydrating paper, and weighed afterwards (*M*_1_). Thawing loss was calculated in accordance with Equation (1):

(1) $$\mathrm{Thawing}\;\mathrm{loss}\;(\%)=\frac{M_{0}-M_{1}}{M_{0}}\times 100$$

### 2.5. Cooking Loss

Cooking loss was determined in accordance with the method described by Faridnia et al. [12]. The thawed samples were weighed (*M*_1_), then cooked in a water at 85 °C until the central temperature of the samples reached 75 °C. The sample was immediately blotted dry and weighed (*M*_2_), and cooking loss was determined in accordance with Equation (2):

(2) $$\mathrm{Cooking}\;\mathrm{loss}\;(\%)=\frac{M_{1}-M_{2}}{M_{1}}\times 100$$

### 2.6. Water Holding Capacity

The water holding capacity of the shrimp samples was derived from the results of Ma et al. [13], adapted slightly. About 10 g of shrimp muscle (*W*_1_) was put in a centrifuge tube with a filtration paper cartridge, then centrifuged at 5000 r·min^−1^ for 15 min at 4 °C. The filter paper was removed, and then the sample was weighed (*W*_2_). Calculations were carried out in accordance with Equation (3):

(3) $$\mathrm{Water}\;\mathrm{holding}\;\mathrm{capacity}\;(\%)=\frac{W_{2}}{W_{1}}\times 100$$

### 2.7. Hardness

Shrimps were deveined, and muscle was taken from the second joint of the abdomen (dimensions: 1.5 × 1.5 × 1.5 cm). The hardness of the shrimp was determined using the TA.XT plus C (Stable Micro Systems, London, UK) mode of a mass spectrometer. Using a P_0.5_ probe, the sample blocks were pressurized at a speed of 1 mm/s to a target variable of 50% relative to the height for testing, and all tests were performed a minimum of ten times.

### 2.8. Color

The a* (red-greenness), b* (yellow-blueness), and L* (brightness) values of thawed and fresh samples (blotting the surface of the samples with filter papers) were surveyed using a colorimeter (CR-20, Konica Minolta Inc., Chiyoda, Tokyo, Japan). The colorimeter needed to be calibrated using its own calibration plate before use [14].

### 2.9. Low-Field Nuclear Magnetic Resonance and Magnetic Resonance Imaging

Low-field nuclear magnetic resonance (LF-NMR) determination was carried out using an NMI 20-060H-I NMR analyzer (Niumag Corporation Ltd., Suzhou, China) following the approach summarized by Zhou et al. [15] with slight modifications. Thawed and fresh shrimps were wrapped in plastic wrapping, and placed in NMR sample tubes. The Q-CPMG mode was selected and the measurement temperature was 32 °C. The samples were subjected to relaxation time, *T*_2_, scans and each scan was repeated four times. This was followed by applying the analysis software accompanying this instrument to perform a batch inversion of the sequence exponential decay profile to obtain the transverse relaxation time, *T*_2_, spectra of the different samples.

After the completion of the Q-CPMG mode test, the sample was analyzed via multi-layer spin-echo pulse sequence imaging using the magnetic resonance imaging (MRI) technique to determine the proton density curve of the samples. The shrimps were wrapped in cling film, and placed in an NMR tube, and the samples were imaged, and *T*_2_-weighted with the following conditions: repetition time (TR) = 1000 ms, and an echo time (TE) = 20 ms. The obtained imaging spectra were mapped using Niumag NMR V3.0 image processing software, and transformed into pseudo-color maps, which allowed the visualization of the moisture distribution of the shrimps.

### 2.10. Light Microscopy Observation

The microstructure of the shrimp was measured following the method of Sun et al. [16], slightly modified. The second segment of abdominal muscle (0.5 cm × 0.5 cm × 1.0 cm) of shrimps was taken and fixed in Carnoy’s solution (10% acetic acid, 30% chloroform, and 60% pure ethanol; *v*/*v*) for 48 h. The samples were then gradually dehydrated in different concentrations of alcohol (50%, 75%, 85%, 95%, and 100%) for 30 min each. The samples were then made transparent with xylene, followed by being submitted to paraffin soaking and embedding. After completing the above steps, the paraffin-embedded tissues were cut perpendicularly into 5 μm thick sections using a microtome, and then stained in hematoxylin–eosin solution. After staining was completed, the sections were rinsed with water for 15 min, dried, and then sealed using resin. At last, the vertical fibrous portions of the shrimp samples were observed with a light microscope (magnification 400×), and photographed with a digital camera.

### 2.11. Scanning Electron Microscopy

Scanning electron microscopy (SEM) images of the sample were observed following the approach outlined by Yang et al. [10] with some modifications. Frozen samples were cut into 2 mm thick slices along the perpendicular orientation of the muscle fibers, after which they were freeze-dried in a vacuum freeze-dryer. The temperature of the condensing sheet was set to −60 °C, and the time was set to 48 h. After drying, the samples were attached to conductive double-sided tape, and the surface was sprayed with a gold-palladium alloy for 50 s using a gold sprayer. A scanning electron microscope (at 100× magnification) was utilized to observe the microstructure of the shrimp samples at an acceleration voltage of 3 kV.

### 2.12. Statistical Analysis

All of experiments were repeated more than three times, and the data obtained were expressed as the mean ± standard deviation. One-way analysis of variance was performed on the data for each indicator using SPSS software (version 28.0, IBM, Chicago, IL, USA). Post hoc multiple comparison (Tukey’s HSD test) was performed to evaluate significant differences between all samples at a level of significance of *p* < 0.05. Figures were plotted using Origin 2023 software (OriginLab, Northampton, MA, USA).

## 3. Results and Discussion

### 3.1. Freezing Curves

**Figure 1:**
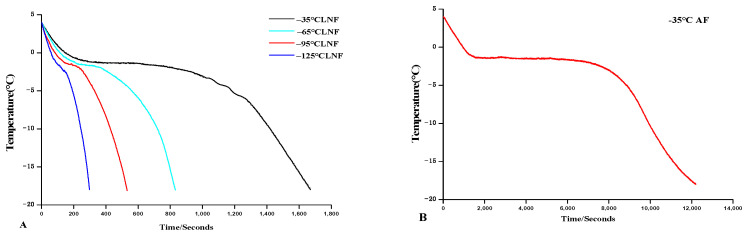
Freezing curves of vannamei shrimps at different LNF temperatures (**A**), and freezing curves of vannamei shrimps at −35 °C AF (**B**).

The freezing curve describes the relationship curve between the temperature of the food and time during the freezing process. Figure 1A represents the freezing curves of shrimp under different LNF temperatures, and Figure 1B represents the freezing curves of shrimp under AF; the total frozen time (4 °C~−18 °C) was significantly different among all the groups (*p* < 0.05). It can be seen from Figure 1 that the freezing speed of LNF groups was faster than that of the AF group. This may be due to the heat transfer coefficient of liquid nitrogen is much higher than that of air [17]. The results of this study are consistent with those of Zhu et al. [18], who found that the freezing speed of wolfberry at different liquid nitrogen temperatures was faster than that using AF. At the same time, the lower the liquid nitrogen temperature was, the shorter the frozen time was. The freezing speed of the −125 °C LNF group was the fastest, and the total freezing time was about one fortieth of that of the AF group, followed by that of the −95 °C LNF, −65 °C LNF, and −35 °C LNF groups (*p* < 0.05). This is because the lower the temperature of liquid nitrogen, the faster the heat exchange with the shrimps. Likewise, Yang et al. [10] also found that the lower the LNF temperature was, the shorter the freezing time was.

### 3.2. Thawing Loss Analysis

**Figure 2:**
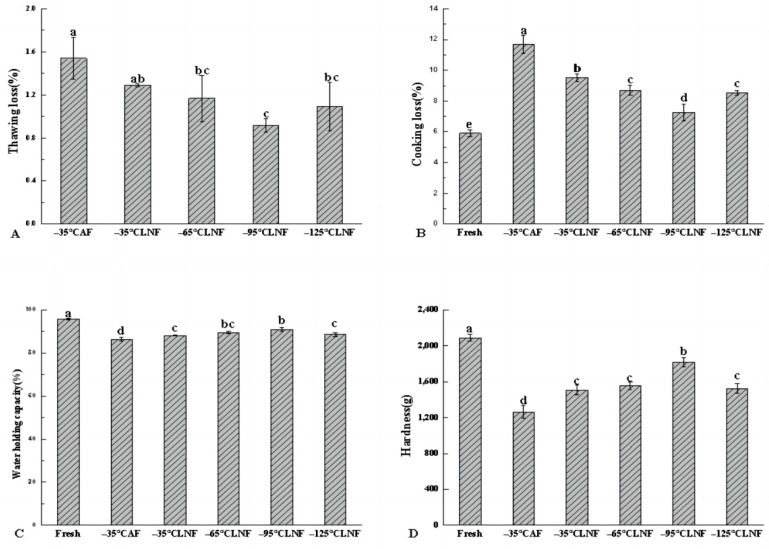
Changes in thawing loss (**A**), cooking loss (**B**), water holding capacity (**C**), and hardness of vannamei shrimps with different frozen treatments (**D**). Different letters indicate a significant difference (*p* < 0.05).

Frozen aquatic products need to be thawed before they can be processed and eaten by people. The process of freezing-thawing is often accompanied by a loss of juice, including water, amino acids, and other nutrients [19]. Therefore, thawing loss is an important index with which to assess the quality of aquatic products after freezing. As shown in Figure 2A, the thawing loss of LNF groups was lower than that of the AF group, which is consistent with the research results of Yang et al. [10]. This maybe because shrimps could quickly cross the biggest ice crystal formation area after LNF, forming small and dense ice crystals in the shrimp, and causing less damage to cells, resulting in less juice loss [20]. However, the freezing speed of the AF group was slow, and large and irregular ice crystals were generated in the cell gap. The muscle damage to the AF shrimp meat was extensive, and prevented water from coming back to the cells after thawing [21], resulting in more loss of juice. Xie et al. [22] discovered that fast-freezing low-voltage-electric-field-assisted freezing reduced the thawing loss of steak compared with that under AF. This study also found that thawing losses decreased with decreasing liquid nitrogen temperatures over a range. Among all the frozen samples, the thawing loss of LNF at −95 °C was the smallest (0.91%), and was significantly different from that of the AF group (1.53%) (*p* < 0.05). In other words, thawing loss under LNF was smaller than that under AF, and −95 °C LNF was the best temperature for LNF.

### 3.3. Cooking Loss Analysis

Cooking loss usually points to a loss of food quality caused by the leakage of water and soluble substances after heating treatment [23]. It also reflects the hydration of protein in muscle under different processing conditions, which is one of the key indicators for evaluating a food’s water holding capacity and quality [24]. Figure 2B shows the cooking loss of shrimps in the fresh, AF, and LNF groups. The cooking loss in the fresh group was the lowest (5.9%), while that of the AF group was the highest (11.69%). The cooking loss of LNF groups was significantly lower than that of the AF group (*p* < 0.05), and this may be due to LNF promoting the formation of smaller ice crystals in shrimp, causing less damage to muscle cells. At the same time, cooking loss is also related to the degree of protein denaturation; the higher the degree of protein denaturation, the greater the cooking loss. This is because in slow freezing, protons concentrate in unfrozen water, causing a decrease in pH near the protein structure, leading to protein denaturation [25]. In rapid freezing, small ice crystals trap protons, and rapid freezing results in a less pronounced denaturation of myofibrillar proteins compared to that under slow freezing. For the LNF groups, the decrease in temperature promoted a decrease in cooking loss, but when the freezing temperature was too low (−125 °C), cooking loss increased. This may be because the temperature difference between the ambient temperature and the shrimp center temperature was too large; the shrimp surface froze rapidly, and the internal water changed phase, causing volume expansion and a pressure rise. The large internal and external pressure differences caused cell damage, which led to the phenomenon of low-temperature cell fracture [8]. As mentioned above, LNF reduced the cooking loss of shrimp, with −95 °C LNF being the best.

### 3.4. Water Holding Capacity Analysis

Water holding capacity points to the ability of meat to keep its own water and add water from the outside [26]; the water content of *Litopenaeus vannamei* is higher, so water holding capacity is also a significant indicator with which to evaluate the quality of *Litopenaeus vannamei*. As shown in Figure 2C, after freezing treatment, the water holding capacity of shrimp decreased compared with that of fresh samples. This may be caused by protein decomposition and muscle contraction, leading to water being squeezed out of cells [27]. The water holding capacity of the fresh group was 95.76%, while that of the AF group was only 86.42%. The water holding capacity of the AF group was lower than that of the LNF groups, and this may be due to the freezing speed of the AF group being slow, resulting in the formation of large and irregular ice crystals, destroying the muscle tissue, thus reducing the water holding capacity. In LNF groups, with the decrease in freezing temperature, the water holding capacity showed a trend of increasing first and then decreasing, with that of the −95 °C LNF group being the highest. This may be due to the fact that compared with the control group, a certain temperature range of LNF (−35 °C~−95 °C) accelerated the speed of shrimp passing through the biggest ice crystal formation area, and resulted in the formation of small and dense ice crystals, thus reducing injury to cells. However, at −125 °C LNF, the water holding capacity of shrimp decreased. This may be due to the low temperature causing the cells to break at low temperatures, decreasing the water holding capacity of shrimp muscle. In conclusion, −95 °C LNF frozen shrimps were the best at maintaining water holding capacity among all the frozen treatments.

### 3.5. Hardness Analysis

Hardness is a vital evaluation index of food quality and an important parameter that affects the acceptability and mechanical processing of seafood products [28]. From Figure 2D, it can be seen that compared to the hardness of the fresh group (2091.62 ± 37.80 g), the hardness of all the frozen shrimp was reduced, whereas the hardness of LNF groups was higher than that of the AF group (*p* < 0.05). This was due to the formation of tiny ice crystals within the muscle of the shrimp during LNF, which caused less cellular destruction, whereas for the AF group, a slower freezing speed caused the formation of larger ice crystals, increasing the destruction of muscle fibers, which led to harder shrimp [29]. The hardness of the −95 °C LNF group was the highest in the LNF groups, while the hardness outcomes of −35 °C, −65 °C, and −125 °C LNF groups were not significantly different from each other (*p* > 0.05). Within a certain temperature interval (−35 °C to −95 °C), the lower the temperature, the smaller the ice crystals formed and the higher the water holding capacity of the cells, resulting in higher hardness values, which is consistent with the results of water holding capacity (Figure 2C). In contrast, after freezing at −125 °C LNF, muscle cells experienced low-temperature fracture, and a higher degree of destruction of the fibrous structure of the muscle, which in turn decreased the hardness values of the shrimp. This result is similar to the findings of Pan et al. [30], who found that 50% of the shrimp muscle cell surface was cracked after LNF at −120 °C; greater alterations in muscle fiber structure cause a decrease in hardness values. From this, we know that the hardness values of shrimps were reduced to varying degrees after freezing treatment, while they were better maintained after freezing at −95 °C LNF.

### 3.6. Color Analysis

Color is also an important factor of aquatic products, and has an important impact on the appearance of aquatic products and their acceptance by consumers [31]. As can be seen from Table 1, the fresh group had lower L* values than the frozen groups did (*p* < 0.05). The decrease in L* values caused by freezing may be due to the fact that during the freezing treatment of shrimps, the intracellular water migrated outwards with the formation of ice crystals, causing the intracellular solute concentration to increase. It has been found that the increase in solute concentration increased light absorption [32], and this is also the reason for the rise in L* of shrimp after the freezing treatment. The L* of the AF group was higher than that of the LNF groups (*p* < 0.05), which may be due to the large ice crystals produced caused by AF promoting the destruction of the cellular structure of shrimp muscle. After thawing, as the cell membrane was exposed to different degrees of damage from ice crystals, intracellular water migrated outward, which led to there being more water on the surface of AF shrimp muscle, and increased the refraction of light, thus increasing the L* value of shrimp. Among all the LNF groups, in a certain temperature interval (−35 °C to −95 °C), the lower the temperature, the less the water migrated to the muscle surface, and thus the lower the L* value, with the lowest L* value at −95 °C, while the L* value under −125 °C LNF was higher than that under the −95 °C LNF. This may be because the freezing speed of −125 °C LNF was too fast, and the large pressure difference between the inside and outside caused some of the cells to break at a low temperature [8], which hindered the reabsorption of outward migration water after thawing, resulting in more water on the surface of shrimp muscle, and a higher L* value. In addition, it can be seen from Table 1 that the a* values were not significantly different between all the groups (*p* > 0.05), and this is consistent with the results of Hafezparast-Moadab et al. [33], who found no significant change in the a* values of rainbow trout fillets at different freezing speeds. Yu et al. [34] thought that the yellow pigment produced due to fat oxidation might increase the b* values. There were no significant differences in the b* values of all samples in the present study (*p* > 0.05), which may be due to non-significant differences in lipid oxidation between freezing treatments. In summary, freezing increased the L* values of shrimps, while the color of −95 °C LNF samples was closer to that of fresh shrimp.

### 3.7. Low-Field Nuclear Magnetic Resonance and Magnetic Resonance Imaging Analysis

**Figure 3:**
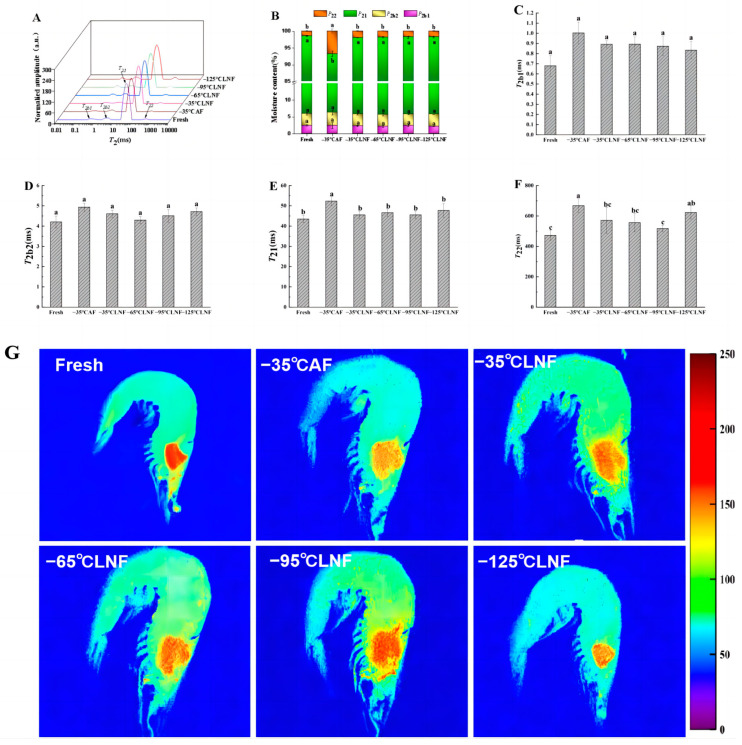
Changes in water properties in vannamei shrimp muscle under different frozen treatments. *T*_2_ relaxation times (**A**); different types of water content (%) (**B**); *T*_2b1_ relaxation times (**C**); *T*_2b2_ relaxation times (**D**); *T*_21_ relaxation times (**E**); *T*_22_ relaxation times (**F**); false-color images obtained via *T*_2_-weighted magnetic resonance imaging (**G**). The velocity bar right shows the correspondence between proton signal intensity and color, the weakening of the proton intensity corresponds to a gradual reddening of the colour of the bands. *P*_2b1_, *P*_2b2_, *P*_21_, and *P*_22_ represent the content of strongly bound water, weakly bound water, immobilized water, and free water, respectively. Different letters in the same index indicate a significant difference (*p* < 0.05).

The LF-NMR technique is a non-destructive detection technique that can rapidly detect the mobility and presence of water in aquatic products [35]. Figure 3A shows that four peaks that represent four different categories of water (*T*_2b1_, *T*_2b2_, *T*_21_, and *T*_22_) in shrimp muscle were found. *T*_2b1_ represents strongly bound water, *T*_2b2_ represents weakly bound water, *T*_21_ represents immobilized water, and *T*_22_ represents free water. The integrated area as a percentage of the total area for the different *T*_2_ intervals is shown as *P*_2_ in Figure 3B. As shown in Figure 3C,D, the *T*_2b1_ and *T*_2b2_ relaxation time was not significant in all samples (*p* > 0.05). This means that there was no significant difference in the mobility of strongly and weakly bound water among all the samples, and these results also correspond to those of *P*_2b1_ and *P*_2b2_ in Figure 3B. This may be due to the fact that bound water is stable and binds tightly to macromolecules such as proteins, and therefore does not migrate to other regions as easily [10].

The states of immobilized and free water changed significantly after different frozen treatments. As shown in Figure 3E,F, the *T*_21_ and *T*_22_ relaxation times of the frozen group were conspicuously longer than those of the fresh samples (*p* < 0.05). This indicates that the freezing treatment reduced the stability of immobilized and free water within shrimp muscle, leading to water migration and loss, resulting in a significant increase in the proportion of immobilized and free water in frozen samples [32]. The *T*_21_ relaxation time in the AF group was conspicuously longer than that in the LNF group, which may have been caused by the greater mechanical damage to the cell membrane or intracellular myofibrillar protein network structure caused by the large ice crystals formed through slow freezing, thus increasing the mobility of water [36]. It can be seen from Figure 3E that the *T*_21_ relaxation times in the LNF groups were not significantly different between each other (*p* > 0.05), indicating that all LNF groups were able to reduce the shifting and loss of the immobilized water. This could be due to the fact that the faster freezing speed of LNF produced smaller ice crystals, thus causing less damage to the muscle. Therefore, the change in immobilized water was not significant in the LNF groups. A similar change can be seen in Figure 3B for *P*_21_. Yang et al. [10] also demonstrated that a shorter *T*_21_ and higher *P*_21_ were obtained in LNF golden pompano compared to those under normal AF. As can be seen in Figure 3F, the *T*_22_ relaxation times of the LNF groups were all shorter than those of the AF group, with the *T*_22_ relaxation time of the −95 °C LNF group being the shortest. This was closer to that of the fresh group. This may be due to the rapid speed of LNF making the water in cells freeze into ice crystals before it migrated outside the cell, resulting in less free water between the muscle fibers [10], whereas the *T*_22_ relaxation time of the −125 °C LNF group was longer, and this may be due to the fact that −125 °C was too low, causing the cells to undergo cryogenic rupture, which caused the intracellular water to transform into more free water. Overall, LNF was more beneficial in retaining water in shrimp muscle than AF was. Among all the different temperature gradients of LNF, −95 °C was more favorable for inhibiting shrimp muscle mobility, and the loss of immobilized and free water.

MRI technology is used to measure the amount of hydrogen protons in aquatic products, and visualize the water distribution in samples via proton-density-weighted imaging [32]. Due to the high moisture content of shrimp muscle, water molecules are the main source of protons, and the signal intensity of imaging depends on the number of protons; the higher the water content in the sample, the stronger the signal intensity of proton density, and the higher the brightness (red) of the MRI image [37]. As illustrated in Figure 3G, the center of the shrimp’s head in the fresh group was uniformly bright red, indicating that the shrimp head has the most hydrogen protons, that is, the highest water content. After freezing treatment, the pseudo-color picture of the shrimp turned yellow gradually, indicating that the shrimp after freezing treatment had different degrees of water loss. The center of the shrimp’s head in the AF group was the lightest, being light yellow. This may be explained by the big ice crystals formed during slow freezing, which caused the greatest damage to cells, and led to the most water loss. With the decrease in LNF temperature, the color of the false-color picture of shrimp deepened. The color of the −95 °C LNF group was redder, indicating that there was more water in −95 °C LNF shrimps than in other frozen samples. This may be attributed to the smaller ice crystals in the −95 °C LNF group, causing less damage to the shrimp muscle, resulting in less water loss. However, the color in the MRI image of the −125 °C LNF group was lighter than that of −95 °C LNF group, indicating that the water content in −95 °CLNF shrimps was less. It may be that too low an LNF temperature led to freezing fracture, resulting in excessive water loss. This is consistent with the results of the previous indicators of water holding capacity. In summary, compared to the fresh group, −95 °C LNF had a redder color and the highest water content in MRI images.

### 3.8. Light Microscopy Analysis

**Figure 4:**
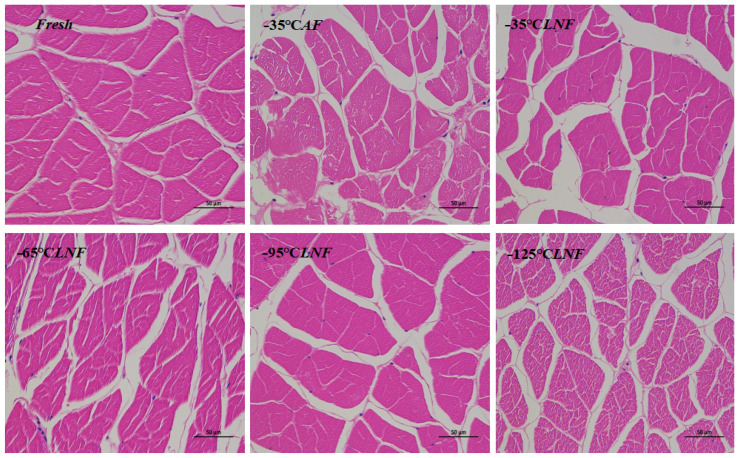
Light microscopy images (magnification: 400×) of vannamei shrimp muscles under different frozen treatments.

Ice crystals formed during the freezing of fish products can affect myogenic fibers, and tissue sections allow the extent of damage to myogenic fibers by ice crystals to be observed relatively clearly under a microscope [38]. As can be seen in Figure 4, the red area represents the muscle tissue, and the white area represents the cytosolic space. From Figure 4, we can clearly observe that the muscle tissue of fresh shrimp was more evenly distributed and regular in shape. Meanwhile, the cell spaces were small. Furthermore, after different freezing treatments, cell membrane rupture increased, and cell spaces were enlarged to different degrees. Compared to the LNF groups, the AF group had more incomplete muscle tissue, blurred muscle bundles, severe cellular rupture, and larger cellular gaps. The reason was that the AF sample were slowly frozen in the air blast freezer, the ice crystals formed between the cells were large, and the ice crystals damaged cellular organization, resulting in the thawing of the extracellular water reabsorbed being impeded, so the cell gaps became larger. In addition to inter-cellular squeezing, this may even puncture the cells, resulting in cell rupture [39]. Compared with the AF group, the muscle bundles in the LNF group remained relatively intact, and only the extracellular gap increased to different degrees, probably because the ice crystals formed inside the shrimp muscle during the LNF process were fine and uniform, which caused less damage to the cells. At the same time, the different temperatures of liquid nitrogen made the ice crystals formed in different sizes, and the degree of cellular damage was also different; the lower the temperature of liquid nitrogen, the smaller the ice crystals formed, and the less damage to cells. In this study, the −95 °C LNF group had the most complete muscle tissue, which was closest to that of the fresh group. This may be due to the smaller ice crystals formed during −95 °C LNF, causing less damage to the cells. Shi et al. [40] also found that the muscle of perch was more complete, and that the membrane gap was smaller under LNF than under AF. In conclusion, −95 °C LNF was more beneficial to the maintenance of the morphology of the muscle tissue of shrimps than other frozen treatments.

### 3.9. Scanning Electron Microscopy Analysis

**Figure 5:**
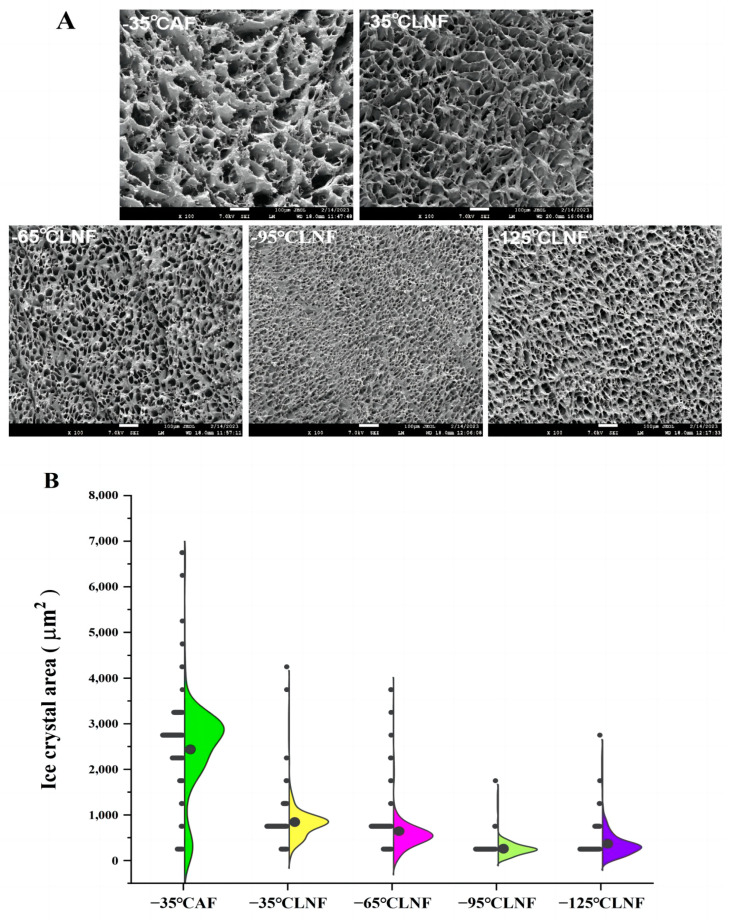
Scanning electron microscopy images (magnification: 100×) of vannamei shrimp muscles with different frozen treatments (**A**); SEM image of ice crystal area distribution statistics (**B**).

Scanning electron microscopy can be used to observe the distribution of pores formed by ice crystals after the freeze-drying of frozen foods [41]. The microscopic structure of shrimp muscle cross-sections after divergent frozen treatments is shown in Figure 5A. The muscle microstructure of the fresh sample without any freezing treatment was relatively complete and dense. Whereas after different freezing treatments, pores of different sizes and diameters were increased between the muscle fibers due to the formation of ice crystals. In general, the size of pores is determined by the size and shape of the ice crystals. The bigger the ice crystals, the bigger the pores. From Figure 5A, we can see that the AF group had the biggest pores among all the frozen samples. This is due to the slower freezing speed under AF, which first started to freeze outside the cell, while the water inside the cell migrated outside due to osmotic pressure, leading to the formation of big ice crystals. The muscle fibers were squeezed by the larger ice crystals that formed around the cells, which manifested themselves as large holes of different pore sizes in SEM images after freeze-drying [39]. In LNF groups, as the liquid nitrogen temperature continued to decrease, the size of the pores showed a decrease (−35 °C to −95 °C), and subsequently an increase (−125 °C), with that of the −95 °C LNF group’s minimum pore size (*p* < 0.05). This may be due to the fact that the lower the LNF temperature, the faster the freezing speed, the finer the ice crystals formed between the cells, and the less damage to the muscle fibers. In contrast, pores increased under −125 °C LNF, probably due to the cryogenic rupture of the cell with the excessively low temperature being used, leading to the water inside the cell flowing outside the cell to form large ice crystals [8].

Figure 5B is a statistical map of the ice crystal area produced after analyzing the SEM images using Image J (version v1.8.0, National Institute of Health, Bethesda, MD, USA) software. About 200 pores were selected for each group of samples, and by numbering and calculating the contour of each pore, the ice crystal area of the frozen samples could be obtained at last [37]. The peaks of the frequency distribution curves in the AF group were relatively smooth, indicating that the ice crystals formed in the AF group were not only larger in area, but also inhomogeneous. In the LNF group, the temperature of liquid nitrogen was progressively reduced, and the area of ice crystals gradually decreased and then increased, which was the same trend as that shown in the SEM plots.

In addition, we performed a correlation analysis between the maximum frequency area of the ice crystals and the hardness values using SPSS software. The Pearson correlation coefficient for both of them was obtained as *p* = −0.803, when the Pearson correlation coefficient was a strong correlation in the interval (0.60~1.00) [42], so the maximum frequency area of the ice crystals and hardness value were negatively and strongly correlated. This indicates that the larger the ice crystal, the smaller the hardness value, and the AF group had the largest ice crystal area, so it had the smaller hardness value. In the LNF groups, −95 °C LNF led to the smallest ice crystal area, and its hardness value was also the largest. In conclusion, −95 °C LNF could better control the size of ice crystals during freezing, comprising a better liquid nitrogen method with which to maintain the muscle hardness of shrimp.

## 4. Conclusions

The effects of different liquid nitrogen temperatures on the freezing speed and muscle quality of shrimps were investigated in the present study. The lower the liquid nitrogen temperature, the faster the freezing speed. The shortest freezing time was achieved when the liquid nitrogen temperature was −125 °C, but muscle under this treatment showed significant microstructural damage. An appropriate liquid nitrogen temperature (−95 °C) diminished thawing losses, cooking losses, and L* values of shrimps, as well as reducing the loss of immobilized and free water in shrimps. In addition, a liquid nitrogen temperature of −95 °C could maintain good water holding capacity and hardness, resulting in smaller microstructural damage. In conclusion, appropriate liquid nitrogen temperatures (−95 °C) accelerated the freezing speed, and improved the muscle quality of frozen shrimp. Therefore, shrimp products can be frozen via spraying with −95 °C liquid nitrogen, thereby realizing the high-quality freezing of aquatic products and improving the market competitiveness of aquatic products.

## Figures and Tables

**Table 1:** Color changes in vannamei shrimps under different frozen treatments.

Treatment	L*	a*	b*
Fresh	28.40 ± 0.78 ^d^	0.47 ± 0.06 ^a^	−0.43 ± 0.06 ^a^
−35 °CAF	37.00 ± 0.92 ^a^	0.70 ± 0.10 ^a^	−0.50 ± 0.10 ^a^
−35 °CLNF	33.83 ± 0.91 ^b^	0.57 ± 0.15 ^a^	−0.47 ± 0.06 ^a^
−65 °CLNF	32.90 ± 0.50 ^b^	0.53 ± 0.15 ^a^	−0.50 ± 0.10 ^a^
−95 °CLNF	30.43 ± 0.76 ^c^	0.53 ± 0.25 ^a^	−0.40 ± 0.10 ^a^
−125 °CLNF	34.23 ± 0.15 ^b^	0.53 ± 0.15 ^a^	−0.57 ± 0.15 ^a^

Different letters in each column indicate a significant difference (*p* < 0.05).

## Data Availability

All relevant data are included in this article.

## References

[1] Zhang B., Fang C.-D., Hao G.-J., Zhang Y.-Y. (2018). Effect of kappa-carrageenan oligosaccharides on myofibrillar protein oxidation in peeled shrimp (*Litopenaeus vannamei*) during long-term frozen storage. Food Chem..

[2] Hsu W.-H., Lai Y.-J., Wu S.-C. (2017). Effects of the anti-microbial peptide pardaxin plus sodium erythorbate dissolved in different gels on the quality of Pacific white shrimp under refrigerated storage. Food Control.

[3] Nakazawa N., Okazaki E. (2020). Recent research on factors influencing the quality of frozen seafood. Fish. Sci..

[4] Pilipovik M., Riverol C. (2013). Reducing Operating Costs in Freezing Processes Using Asymmetric Distribution of Sprays. J. Food Process. Eng..

[5] Ketata M., Desjardins Y., Ratti C. (2013). Effect of liquid nitrogen pretreatments on osmotic dehydration of blueberries. J. Food Eng..

[6] Hu L., Ying Y., Zhang H., Liu J., Chen X., Shen N., Li Y., Hu Y. (2021). Advantages of liquid nitrogen freezing in long-term frozen preservation of hairtail (*Trichiurus haumela*): Enzyme activity, protein structure, and tissue structure. J. Food Process Eng..

[7] Xu Y., Song M., Xia W., Jiang Q. (2019). Effects of freezing method on water distribution, microstructure, and taste active compounds of frozen channel catfish (*Ictalurus punctatus*). J. Food Process Eng..

[8] Lv Y., Chu Y., Zhou P., Mei J., Xie J. (2021). Effects of Different Freezing Methods on Water Distribution, Microstructure and Protein Properties of Cuttlefish during the Frozen Storage. Appl. Sci..

[9] Teng X., Liu Y., Chen L., Xue C., Li Z. (2023). Effects of liquid nitrogen freezing at different temperatures on the quality and flavor of Pacific oyster (*Crassostrea gigas*). Food Chem..

[10] Yang Z., Liu S., Sun Q., Zheng O., Wei S., Xia Q., Ji H., Deng C., Hao J., Xu J. (2022). Insight into muscle quality of golden pompano (*Trachinotus ovatus*) frozen with liquid nitrogen at different temperatures. Food Chem..

[11] Atani S.H., Hamdami N., Dalvi-Isfahan M., Soltanizadeh N., Fallah-Joshaqani S. (2022). Effects of microwave-assisted freezing on the quality attributes of lamb meat. Int. J. Refrig..

[12] Faridnia F., Ma Q.L., Bremer P.J., Burritt D.J., Hamid N., Oey I. (2015). Effect of freezing as pre-treatment prior to pulsed electric field processing on quality traits of beef muscles. Innov. Food Sci. Emerg. Technol..

[13] Ma X., Mei J., Xie J. (2021). Effects of multi-frequency ultrasound on the freezing rates, quality properties and structural characteristics of cultured large yellow croaker (*Larimichthys crocea*). Ultrason. Sonochem..

[14] Jia N., Kong B., Liu Q., Diao X., Xia X. (2012). Antioxidant activity of black currant (*Ribes nigrum* L.) extract and its inhibitory effect on lipid and protein oxidation of pork patties during chilled storage. Meat Sci..

[15] Zhou J., Dong X., Kong B., Sun Q., Ji H., Liu S. (2023). Effects of magnetic field-assisted immersion freezing at different magnetic field intensities on the muscle quality of golden pompano (*Trachinotus ovatus*). Food Chem..

[16] Sun Q., Zhang H., Yang X., Hou Q., Zhang Y., Su J., Liu X., Wei Q., Dong X., Ji H. (2022). Insight into muscle quality of white shrimp (*Litopenaeus vannamei*) frozen with static magnetic-assisted freezing at different intensities. Food Chem. X.

[17] George R. (1993). Freezing proceseses used in the food industry. Trends Food Sci. Technol..

[18] Zhu Z., Luo W., Sun D.-W. (2020). Effects of liquid nitrogen quick freezing on polyphenol oxidase and peroxide activities, cell water states and epidermal microstructure of wolfberry. LWT.

[19] Tironi V., de Lamballerie M., Le-Bail A. (2010). Quality changes during the frozen storage of sea bass (*Dicentrarchus labrax*) muscle after pressure shift freezing and pressure assisted thawing. Innov. Food Sci. Emerg. Technol..

[20] Kaale L.D., Eikevik T.M., Bardal T., Kjorsvik E. (2013). A study of the ice crystals in vacuum-packed salmon fillets (*Salmon salar*) during superchilling process and following storage. J. Food Eng..

[21] Sun Q., Zhao X., Zhang C., Xia X., Sun F., Kong B. (2019). Ultrasound-assisted immersion freezing accelerates the freezing process and improves the quality of common carp (*Cyprinus carpio*) at different power levels. LWT.

[22] Xie Y., Zhou K., Chen B., Wang Y., Nie W., Wu S., Wang W., Li P., Xu B. (2021). Applying low voltage electrostatic field in the freezing process of beef steak reduced the loss of juiciness and textural properties. Innov. Food Sci. Emerg. Technol..

[23] Dalvi-Isfahan M., Hamdami N., Le-Bail A. (2016). Effect of freezing under electrostatic field on the quality of lamb meat. Innov. Food Sci. Emerg. Technol..

[24] Zorzi K., Bonilha S., Queiroz A., Branco R., Sobrinho T., Duarte M. (2013). Meat quality of young Nellore bulls with low and high residual feed intake. Meat Sci..

[25] Zhang Y., Ertbjerg P. (2019). On the origin of thaw loss: Relationship between freezing rate and protein denaturation. Food Chem..

[26] Lund M.N., Luxford C., Skibsted L.H., Davies M.J. (2008). Oxidation of myosin by haem proteins generates myosin radicals and protein cross-links. Biochem. J..

[27] Huff-Lonergan E., Lonergan S.M. (2005). Mechanisms of water-holding capacity of meat: The role of postmortem biochemical and structural changes. Meat Sci..

[28] Lu H., Wang H., Luo Y. (2017). Changes in protein oxidation, water-holding capacity, and texture of bighead carp (*Aristichthys nobilis*) fillets under chilled and partial frozen storage. J. Aquat. Food Prod. Technol..

[29] Leygonie C., Britz T.J., Hoffman L.C. (2012). Impact of freezing and thawing on the quality of meat: Review. Meat Sci..

[30] Pan B.S., Yeh W.-T. (1993). Biochemical and morphological changes in grass shrimp (*Penaeus monodon*) muscle following freezing by air blast and liquid nitrogen methods. J. Food Biochem..

[31] da Silva M.C.A., Leite J.S.F., Barreto B.G., Neves M.V.d.A., Silva A.S., de Viveiros K.M., Passos R.S.F.T., Costa N.P., da Silva R.V., Cavalheiro C.P. (2021). The impact of innovative gluten-free coatings on the physicochemical, microbiological, and sensory characteristics of fish nuggets. LWT.

[32] Cheng S., Wang X., Li R., Yang H., Wang H., Wang H., Tan M. (2019). Influence of multiple freeze-thaw cycles on quality characteristics of beef semimembranous muscle: With emphasis on water status and distribution by LF-NMR and MRI. Meat Sci..

[33] Hafezparast-Moadab N., Hamdami N., Dalvi-Isfahan M., Farahnaky A. (2018). Effects of radiofrequency-assisted freezing on microstructure and quality of rainbow trout (*Oncorhynchus mykiss*) fillet. Innov. Food Sci. Emerg. Technol..

[34] Yu L., Lee E., Jeong J., Paik H., Choi J., Kim C. (2005). Effects of thawing temperature on the physicochemical properties of pre-rigor frozen chicken breast and leg muscles. Meat Sci..

[35] Li F., Zhong Q., Kong B., Wang B., Pan N., Xia X. (2020). Deterioration in quality of quick-frozen pork patties induced by changes in protein structure and lipid and protein oxidation during frozen storage. Food Res. Int..

[36] Sun Q., Sun F., Xia X., Xu H., Kong B. (2019). The comparison of ultrasound-assisted immersion freezing, air freezing and immersion freezing on the muscle quality and physicochemical properties of common carp (*Cyprinus carpio*) during freezing storage. Ultrason. Sonochem..

[37] Zhou J., Sun Q., Wei S., Wang Z., Xia Q., Han Z., Liu Y., Liu S. (2023). Changes in microstructure, quality and water distribution of golden pompano (*Trachinotus ovatus*) muscles subjected to magnetic field-assisted immersion freezing during long-term frozen storage. J. Food Eng..

[38] Tan M., Xie J. (2021). Exploring the Effect of Dehydration on Water Migrating Property and Protein Changes of Large Yellow Croaker (*Pseudosciaena crocea*) during Frozen Storage. Foods.

[39] Alizadeh E., Chapleau N., de Lamballerie M., Le-Bail A. (2007). Effect of different freezing processes on the microstructure of Atlantic salmon (Salmo salar) fillets. Innov. Food Sci. Emerg. Technol..

[40] Shi L., Yang T., Xiong G., Li X., Wang X., Ding A., Qiao Y., Wu W., Liao L., Wang L. (2018). Influence of frozen storage temperature on the microstructures and physicochemical properties of pre-frozen perch (*Micropterus salmoides*). LWT.

[41] Luan L., Wang L., Wu T., Chen S., Ding T., Hu Y. (2018). A study of ice crystal development in hairtail samples during different freezing processes by cryosectioning versus cryosubstitution method. Int. J. Refrig..

[42] Duan W., Qiu H., Htwe K.K., Wang Z., Liu Y., Wei S., Xia Q., Sun Q., Han Z., Liu S. (2023). Correlation between Water Characteristics and Gel Strength in the Gel Formation of Golden Pompano Surimi Induced by Dense Phase Carbon Dioxide. Foods.

